# MS-EmoBoost: a novel strategy for enhancing self-supervised speech emotion representations

**DOI:** 10.1038/s41598-025-94727-2

**Published:** 2025-07-01

**Authors:** Hongchen Song, Long Zhang, Meixian Gao, Hengyuan Zhang, Thomas Hain, Linlin Shan

**Affiliations:** 1 College of Computer and Information Engineering, Tianjin Normal University, Tianjin, 300387 China; 2 School of Computer Science, The University of Sheffield, Sheffield, UK; 3 College of Fine Arts and Design, Tianjin Normal University, Tianjin, 300387 China

**Keywords:** Electrical and electronic engineering, Computer science, Acoustics

## Abstract

Extracting richer emotional representations from raw speech is one of the key approaches to improving the accuracy of Speech Emotion Recognition (SER). In recent years, there has been a trend in utilizing self-supervised learning (SSL) for extracting SER features, due to the exceptional performance of SSL in Automatic Speech Recognition (ASR). However, existing SSL methods are not sufficiently sensitive in capturing emotional information, making them less effective for SER tasks. To overcome this issue, this study proposes MS-EmoBoost, a novel strategy for enhancing self-supervised speech emotion representations. Specifically, MS-EmoBoost uses the deep emotional information from Melfrequency cepstral coefficient (MFCC) and spectrogram as guidance to enhance the emotional representation capabilities of self-supervised features. To determine the effectiveness of our proposed approach, we conduct a comprehensive experiment on three benchmark speech emotion datasets: IEMOCAP, EMODB, and EMOVO. The SER performance is measured by weighted accuracy (WA) and unweighted accuracy (UA). The experimental results show that our method successfully enhances the emotional representation capability of wav2vec 2.0 Base features, achieving competitive performance in SER tasks (IEMOCAP:WA,72.10%; UA,72.91%; EMODB:WA,92.45%; UA,92.62%; EMOVO:WA,86.88%; UA,87.51%), and proves effective for other self-supervised features.

## Introduction

Speech is one of the most common and direct forms of human communication, containing rich semantic and emotional information. Speech Emotion Recognition (SER) technology enables machines to focus on the non-textual aspects of speech, uncovering the latent emotions in speech signals, thereby enhancing the machine’s emotional understanding and abilities to empathize. Currently, SER technology has been widely applied in various fields such as intelligent customer service^1^, health monitoring^2^, and educational teaching^3^  demonstrating significant practical value. However, the accuracy of SER can be influenced by many external factors, including but not limited to individual differences between speakers^4^, methods of extracting emotional features^5^, and the construction of recognition models^6^. These factors make accurate SER a highly challenging task.

In the early stages of SER research, scholars employed a series of computations and transformations on raw speech signals to derive artificially designed acoustic emotion features, such as prosodic and spectral features. These features were combined with traditional machine learning classifiers such as Gaussian Mixture Models (GMM)^7^, Support Vector Machines (SVM)^8^, and Hidden Markov Models (HMM)^9^ to complete SER tasks. With the advent of deep learning technologies, researchers began employing Convolutional Neural Networks (CNNs)^10^, Long Short-Term Memory networks (LSTMs)^11^, and Attention Mechanisms^12^ to extract deep emotional representations from either handcrafted features or directly from raw speech waveforms^13^. However, these approaches typically rely on extensive data annotation and necessitate the development of specialized models tailored for specific SER tasks and application scenarios^14^. In the context of languages or dialects with limited annotated data, the supervised learning method encounters significant challenges.

**Figure 1:**
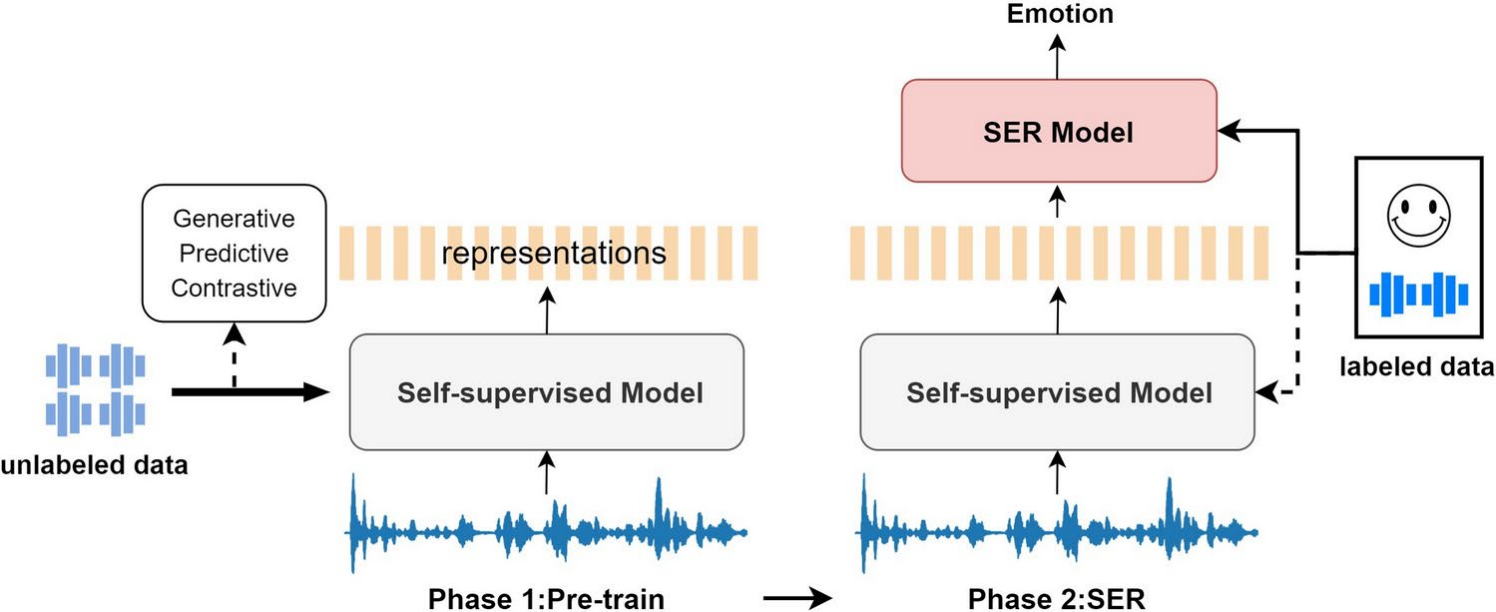
Framework for using self-supervised representation learning in speech emotion recognition.

In recent years, researchers have proposed self-supervised representation learning methods to address the challenges mentioned above. Figure 1 illustrates the application process of self-supervised representation learning in the SER task. In the first phase, the self-supervised model utilizes unlabeled audio data combined with generative, contrastive, and predictive learning methods to acquire high-quality speech representations. In the second phase, the SER task either employs the learned representations from the frozen model or fine-tunes the entire pre-trained model using labeled audio data. The generative method^15^ enables the model to generate or reconstruct data from inputs, thereby facilitating the learning of the intrinsic structures and patterns within the data. Conversely, the contrastive method^16^ strengthens the relationships between similar samples and distinguishes dissimilar ones through the comparison of various data samples. Meanwhile, the predictive method^17^ focuses on comprehending the dynamics of the data by predicting specific characteristics or future states. Prominent self-supervised models such as wav2vec 2.0^16^ and HuBERT^17^ were initially designed to optimize the performance of speech recognition systems. Although the feature embeddings extracted by these models contain rich semantic information, their expression of emotional information is not prominent enough. When using either the last frame or the average of all frames as features for SER tasks, sequence-level features tend to be lost^18^.

To address the aforementioned issues, some researchers have proposed fine-tuning self-supervised pre-trained models^19,20^ to enrich the emotional content within feature embeddings, making them more suitable for SER tasks. Another type of approach involves supplementing self-supervised speech features with additional emotional information from other modalities^21^ or acoustic features^22^. However, these solutions either focus on the model level, involving fine-tuning pre-trained models, or on feature fusion, integrating different modalities or types of acoustic features, without exploring the self-supervised features themselves. Therefore, our work revolves around enhancing the acoustic emotional information within self-supervised features themselves.

Existing speech enhancement technologies provide valuable insights into our work, even as they focus on improving speech quality and intelligibility in noisy environments. Jannu et al.^23^ have highlighted that an effective speech enhancement system relies on accurately modeling the long-term dependencies of noisy speech. Alongside utilizing Transformers for parallel processing, the system incorporates CNN to extract local information. Vanambathina et al.^24^ emphasized the importance of time-frequency (T-F) details and utilized a time-frequency attention (TFA) mechanism to capture significant T-F distributions of speech. Additionally, Jannu et al.^25^ implemented attention mechanisms to focus the model on semantically relevant and critical parts of the original waveform, and added two layers of Gated Recurrent Unit (GRU) at the bottleneck of the encoder-decoder architecture to represent the correlations between adjacent noisy speech frames. Inspired by these studies, we have conducted a comprehensive analysis of self-supervised learning (SSL) features and meticulously assessed the potential contributions of attention mechanisms, CNNs, and other technologies in our work. We considered both the long-term dependencies and T-F details of speech to enhance self-supervised speech emotion representations. Our main contributions are as follows:

- We propose MS-EmoBoost, a novel strategy for enhancing self-supervised speech emotion representations, which effectively utilizes the deep emotional information in MFCC and spectrogram to enhance self-supervised features.
- Experiments on the IEMOCAP, EMODB, and EMOVO datasets have demonstrated that our method effectively enhances the feature representation of the wav2vec 2.0 Base model.
- We prove that the MS-EmoBoost strategy is generalizable across various self-supervised feature extraction scenarios.

## Related work

To ensure that speech emotion features accurately capture both the long-term dependencies of speech and T-F details, we have conducted an in-depth analysis of self-supervised features, examining their strengths and limitations. To address these limitations, we further explored additional acoustic features beneficial to our research and decided to robustly extract emotional information from these features to guide the enhancement of self-supervised features. In this section, we will review the development of SER, discuss the characteristics of self-supervised features, and introduce some related literature in acoustic feature selection and model construction.

The emotions of a speaker often influence the production of speech signals^26^, and hence the characteristics of a speech signal can to some extent reflect the speaker’s emotional state. Inspired by this theory, researchers have utilized temporal and spectral algorithms to design three major types of acoustic features regarding speech emotion: prosodic features^27^, timbral features^28^, and spectral features^29^. These respectively capture the rhythm and pitch variations, timbre and sound quality, as well as the intensity and distribution of frequencies in speech signals. To capture the nonlinear relationships in speech signals, researchers have employed deep learning technologies to extract deep emotional features from either handcrafted acoustic features or raw speech waveforms. These features have made substantial contributions to the field of SER.

Stacked Transformer^30^ layers, as the core components of self-supervised models such as wav2vec 2.0 and HuBERT, can effectively model the contextual information of audio, thus adeptly capturing the long-distance dependencies within audio sequences. In contrast, these models demonstrate a somewhat limited ability to capture T-F details in speech. Therefore, utilizing spectral features rich in T-F details to enhance the emotional representation capabilities of these self-supervised models is a viable strategy.

Spectrogram is a visual representation of audio signals in time and frequency domains, typically depicted in varying colors or shades to indicate the intensity of spectral components. Beyond containing rich T-F information, spectrogram also encapsulates substantial emotional information. To effectively extract these details, researchers have undertaken extensive explorations and efforts. Zheng et al.^31^ focused on the differences in emotional expression among various spectrograms and constructed a Deep Convolutional Neural Network (DCNN) to learn representations of emotions from labeled training data segments. To maintain robust emotional recognition performance in complex scenarios, Huang et al.^32^ introduced a Semi-supervised Convolutional Neural Network (Semi-CNN) to learn salient emotional features from spectrograms. Wani et al.^33^ proposed the Deep Stride Convolutional Neural Network (DSCNN), which maintaining recognition accuracy while enhancing the computational speed of the model.

Mel frequency cepstral coefficient (MFCC) simulate the auditory characteristics of the human cochlea. They are derived from further processing of spectral information, thereby preserving essential information within the frequency domain and generating a set of feature parameters that are easier to handle and differentiate. Kumbhar et al.^34^ conducted preliminary investigations into emotion recognition from MFCC features using the LSTM algorithm, demonstrating its effectiveness in extracting deep features from MFCC. Bhandari et al.^11^ explored the impact of LSTM hidden layer size and output dimensions on extracting emotional information from MFCC features, presenting the practical implementation of an appropriate LSTM model in a SER system. Concurrently, Wang et al.^35^ considered the latent emotional information in both mel-spectrogram and MFCC, employing a standard LSTM to process MFCC features and proposing a Dual-Sequence LSTM (DS-LSTM) to handle mel-spectrograms, jointly predicting the emotional category of speech.

Attention Mechanism assists models in identifying key frames within speech signals, thereby enhancing their emotional perception capabilities. Zhou et al.^36^ extracted multiplexed acoustic information, including visual representations of spectrograms and MFCC from audio signals and employed an attention mechanism to fuse the most salient information from both types of features to accomplish SER task. Li et al.^37^ used the self-attention mechanism to focus on emotionally significant segments within speech, utilizing gender classification as an auxiliary task to address the SER issue. Sun et al.^38^ proposed a novel MCSAN network that integrates the self-attention module with the cross-attention module, effectively merging emotional information from both speech and text. Fu et al.^39^ introduced a new cross-modal fusion network based on self-attention and residual structures, CFN-SR, ensuring the efficient complementarity and integrity of emotional information from both audio and video. Naderi et al.^40^ proposed an attention-based method for effectively fusing wav2vec 2.0 transformer blocks with prosody features, utilizing transfer learning to significantly improve the accuracy of Cross-corpus SER (CCSER).

## Proposed method

**Figure 2:**
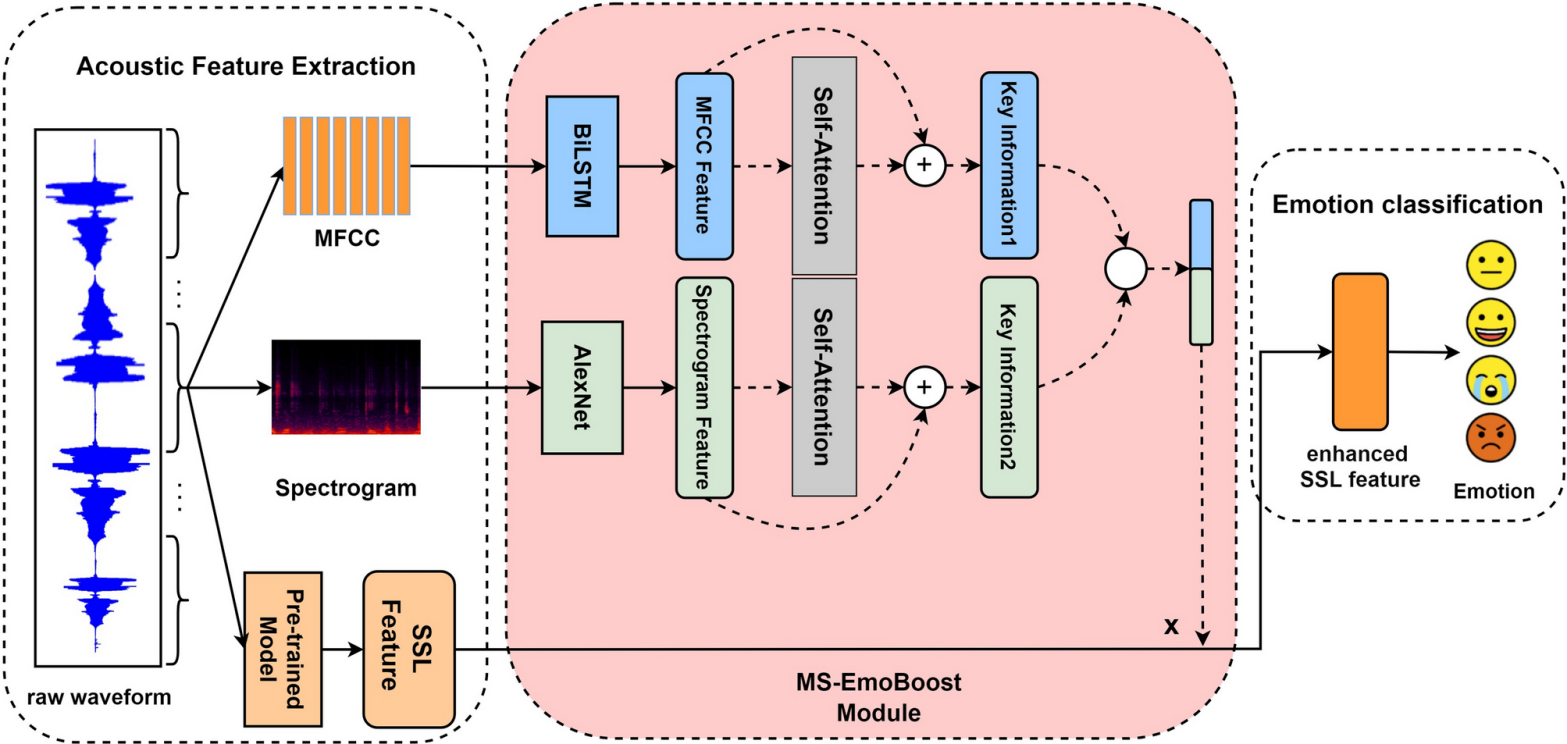
Proposed model structure.

In this section, we introduce MS-EmoBoost, emphasizing how the system enhances emotional information with self-supervised feature extraction. As illustrated in Fig. 2, the proposed model is divided into three main components: acoustic feature extraction, self-supervised feature enhancement, and the final emotion recognition. Each of these components will be discussed in detail in subsequent subsections.

### Acoustic feature extraction

The acoustic feature extraction component is designed to extract MFCC features, spectrograms, and self-supervised features for subsequent feature enhancement tasks.In the following formulation, the original speech waveform is denoted as $x_w \in \mathbb {R}^{T_w \times 1}$, and the aforementioned features are all derived from this original speech waveform.

The MFCC feature extraction initially involves the pre-emphasis of the input speech signal, which aims to enhance the high-frequency components of the signal. Subsequently, the signal is segmented into multiple frames, and a window function is applied to each frame to reduce edge effects. Then, each frame signal is transformed from the time domain to the frequency domain with Fast Fourier Transform (FFT). In the frequency domain, the spectrum is processed by a filter bank based on the Mel scale, which simulates the human ear’s sensitivity to different frequencies. The log energies of the filter outputs are then compressed through the Discrete Cosine Transform (DCT), ultimately yielding the MFCC features, denoted by $x_m \in \mathbb {R}^{T_m \times D_m}$.

The feature extraction process for spectrograms is similar to that of MFCC feature. It begins with some pre-processing steps including pre-emphasis, framing, and windowing of the speech signal to prepare for further analysis. After pre-processing, the Short-Time Fourier Transform (STFT) is applied to each windowed frame, resulting in a matrix that encapsulates both time and frequency information. Subsequently, the logarithm of the amplitude spectrum is calculated and the results are normalized. This enhances the visibility of low amplitude frequencies and compresses the dynamic range, accentuating subtle energy variations. Finally, the required frequency components are extracted and the data shape is adjusted to produce the spectrograms, denoted as $x_s \in \mathbb {R}^{T_s \times D_s}$.

**Figure 3:**
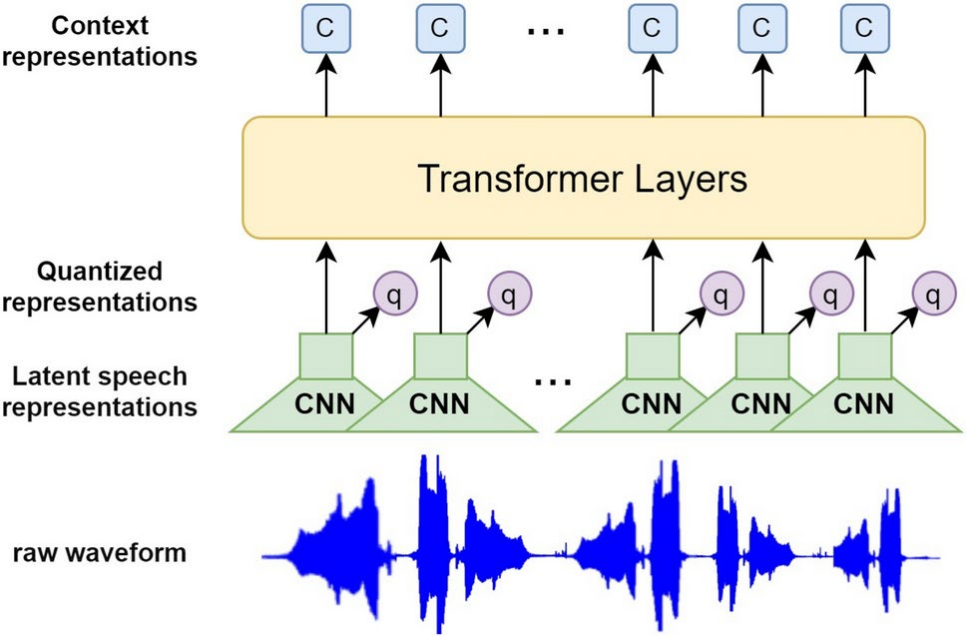
Overview of wav2vec2.0.

As illustrated in Fig. 3, the wav2vec 2.0 architecture employs a self-supervised learning framework, specifically designed to learn speech representations from raw audio waveforms. The process begins with the extraction of latent speech features through multiple layers of CNNs. These latent representations are then partially masked and the masked representations are subsequently input into Transformer layers, which are designed to capture the contextual information of the audio data. By integrating masking techniques with contrastive learning methods, the model is capable of accurately identifying the true latent speech representations from a set of quantized representations associated with the masked time steps. In this study, we utilize wav2vec 2.0 Base^16^, which has been pretrained on the LibriSpeech (LS-960) dataset^41^, to extract self-supervised features from the raw audio waveforms.

1 $$\begin{aligned} X_w = \text {wav2vec 2.0 Base}(x_w) \end{aligned}$$

where $$X_w \in \mathbb {R}^{T'_w \times D'_w}$$.

### MS-EmoBoost module

The MS-EmoBoost Module utilizes key emotional information from MFCC features and spectrograms as guidance to highlight the emotional content within the wav2vec 2.0 Base features, while also incorporating certain frequency domain information to enhance the emotional representation capabilities of the self-supervised features. Figure 2 illustrates the structure and enhancement details of the MS-EmoBoost module.

Firstly, we need to extract the deep emotional representations $X_m$ and $X_s$ from MFCC features and spectrograms, respectively. The deep emotional representations of MFCC features are extracted with a Bi-LSTM with a dropout of 0.5. The deep emotional representations of the spectrograms are processed by the pretrained AlexNet^42^, which has demonstrated excellent performance in the field of computer vision (CV). The specific computation process is formulated as follows

2 $$\begin{aligned} X_m&= \text {Bi-LSTM}(x_m) \end{aligned}$$

3 $$\begin{aligned} X_s&= \text {AlexNet}(x_s) \end{aligned}$$

where $$X_m \in \mathbb {R}^{T'_m \times D'_m}$$, $$X_s \in \mathbb {R}^{T'_s \times D'_s}$$.

Secondly, we have designed a self-attention layer based on a residual structure. The configuration of the self-attention layer is intended to extract more critical emotional information from the deep emotional representations of both MFCC features and spectrograms. Meanwhile, the incorporation of the residual structure aims to prevent the loss of original feature information. The computation process is as follows

4 $$\begin{aligned} X'_m&= X_m + \text {Attention}(Q_m, K_m, V_m) \end{aligned}$$

5 $$\begin{aligned}&\quad \text {Attention}(Q_m, K_m, V_m) = \text {softmax}\left( \frac{Q_m K_m^T}{\sqrt{d_{K_m}}}\right) V_m \end{aligned}$$

6 $$\begin{aligned} X'_s&= X_s + \text {Attention}(Q_s, K_s, V_s) \end{aligned}$$

7 $$\begin{aligned}&\quad \text {Attention}(Q_s, K_s, V_s) = \text {softmax}\left( \frac{Q_s K_s^T}{\sqrt{d_{K_s}}}\right) V_s \end{aligned}$$

where $$X'_m \in \mathbb {R}^{T'_m \times D'_m}$$, $$X'_s \in \mathbb {R}^{T'_s \times D'_s}$$.

In this setup, $X'_m$ and $X'_s$ represent the MFCC features and spectrogram features post-attention application, respectively. $d_{K_m}$ and $d_{K_s}$ denote the embedding dimensions for these features. The calculation methods for $Q_m$, $K_m$, and $V_m$ are provided here (which also applies to $Q_s$, $K_s$, $V_s$).

8 $$\begin{aligned} Q_m = W_o X_m + b_a^Q \end{aligned}$$

9 $$\begin{aligned} K_m = W_k X_m + b_a^K \end{aligned}$$

10 $$\begin{aligned} V_m = W_v X_m + b_a^V \end{aligned}$$

where $W$ and $b$ represent the weight matrices and bias vectors.

Following the attention process, the modified features $X'_m$ and $X'_s$ are flattened and go through a dropout operation (dropout rate = 0.1) to mitigate the risk of overfitting. Considering that both MFCC and spectrogram contain rich emotional guidance information, we employ linear layers equipped with ReLU activation function to project each onto a unified 128-dimensional space, followed by concatenation. Subsequently, in order to derive the feature enhancement matrix $X_{enh}$, the concatenated features are projected into a 149-dimensional space, followed by the application of a dimensional reshaping to facilitate subsequent enhancement processes, referred as $f_{enh}$

11 $$\begin{aligned} X_{\text {enh}} = f_{\text {enh}}(linear_m(X'_m) \oplus linear_s(X'_s)) \end{aligned}$$

where $$X_{\text {enh}} \in \mathbb {R}^{1 \times T'_w}$$.

Finally, we multiply the self-supervised features extracted from the wav2vec 2.0 Base with the feature enhancement matrix to obtain the enhanced self-supervised features. This operation integrates the key information captured by the feature enhancement matrix with the acoustic representations from wav2vec 2.0 BASE^16^, resulting in a stronger representation for emotion recognition task. The computation can be described as follows

12 $$\begin{aligned} X'_w = X_{\text {enh}} \cdot X_w \end{aligned}$$

where $$X'_w \in \mathbb {R}^{1 \times D'_w}$$.

### Emotion classification

We reshape the enhanced self-supervised features $X'_w$ and employ a simple linear layer to complete the final task of emotion classification. The enhanced features are projected onto a 4-dimensional space to match the four emotion categories. The final predictions, $\hat{y}$, are generated using the softmax function, and the computation proceeds as follows

13 $$\begin{aligned} \hat{y} = \text {softmax}(\text {linear}(X'_w)) \end{aligned}$$

We employ the cross-entropy loss, which is widely used in classification tasks, as the loss function for this work. It measures the discrepancy between the model’s predicted probability distribution of emotions and the true label distribution. The formula for cross-entropy loss can be formulated as follows

14 $$\begin{aligned} L = L_{\text {ce}}(y - \hat{y}) \end{aligned}$$

where $y$ is groundtruth.

## Experiment

### Dataset

The first database, the Interactive Emotional Dyadic Motion Capture (IEMOCAP)^43^, is an English emotional speech database. It includes 12 hours of audio-visual data and text transcription data, recorded by ten actors (five males and five females) with scripted and improvised scenarios. The emotional annotations were independently provided by multiple annotators. In this study, we utilize all audio data recorded in both scripted and improvised scenarios. Following previous studies^18,37^, we merged the “excited” category into the “happy” category and focuses on identifying four emotion categories: “angry (1,103)”, “sad (1,084)”, “happy (1,636)”, and “neutral (1,708)”, which sums up to 5,531 acoustic utterances. We use a 10-fold leave-one-speaker-out(LOSO) cross-validation strategy to assess the effectiveness of our method.

The second database, the Berlin Database of Emotional Speech (EMODB)^44^, is a German emotional speech database. It was recorded by ten native German experts (five males and five females), comprising a total of 535 sentences designed to simulate everyday communication scenarios. The audio data encompasses seven categories of emotions (“angry”, “boredom”, “disgust”, “fear”, “happy”, “neutral”, and “sad”).In this study, we utilize all audio data from EMODB and use a 10-fold LOSO cross-validation strategy to evaluate the performance of our method in recognizing these seven emotional categories.

The third database, EMOVO^45^, is an Italian emotional speech dataset. It contains audio recordings from six native Italian speakers (three males and three females), encompassing a total of 588 utterances designed to reflect a range of emotional states. The database includes seven emotion categories: “anger”, “disgust”, “fear”, “joy”, “neutral”, “sadness”, and “surprise”. In this study, we utilize all available audio data from EMOVO and evaluate our method’s performance in recognizing these emotions using a 10-fold cross-validation strategy.

### Experimental setup

In this study, we sample the acoustic utterances in the datasets at a rate of 16 kHz. Each audio segment is spilt into 3-second clips, with zero-padding employed to fill any segments that are shorter than 3 seconds. Our objective is to predict the emotional state of each audio segment. The emotional state of the entire acoustic utterance is determined by the average of the predictions from all its constituent segments. We employ Librosa^46^ to extract 40-dimensional MFCC features in HTK style. During the extraction of the spectrogram, we use a 40-millisecond Hamming window with a hop size of 10 milliseconds, where each windowed block is treated as a frame. The length of the Discrete Fourier Transform (DFT) is set to 800, and the first 200 DFT points are selected as the required frequency components. Consequently, each audio segment corresponds to a 300 * 200 pixel spectrogram.

The proposed framework is implemented using PyTorch (version 1.10.1). All the experiments are conducted on an Nvidia RTX 3090 GPU. Considering the characteristics of various datasets, the parameter scale of the self-supervised models, and the size of the pre-training data, we appropriately adjusted certain hyperparameters, such as the number of epochs, batch size, and early stopping patience to account for their potential impact on the experimental results. The overall description of the hyperparameters utilized in this work is highlighted in Table 1.

**Table 1:** Hyperparameters employed for this study.

Hyperparameter	Value
Number of epochs	100/150
Learning rate	1e-5
Activation function	ReLU
Dropout rate	0.1
Optimizer	Adam
Loss function	Cross entropy
Batch size	64/32/16
Early stopping patience	8/20

### Evaluation metrics

To comprehensively evaluate our approach, we employ both Weighted Accuracy (WA) and Unweighted Accuracy (UA) to evaluate the model’s performance across different emotion categories. WA considers the number of samples in each category within the dataset, assigning greater weight to categories with larger sample sizes, thus adjusting their impact on the overall accuracy. In contrast, UA treats all categories equitably, assessing the model’s overall performance by calculating the average accuracy across various emotional categories. This approach ensures a fair evaluation of all categories, making it especially suitable for situations with imbalanced categories. The computation for WA and UA can be described as follows

15 $$\begin{aligned} \text {WA}&= \frac{\sum _{i=1}^k n_i}{\sum _{i=1}^k N_i} \end{aligned}$$

16 $$\begin{aligned} \text {UA}&= \frac{1}{k} \sum _{i=1}^k \frac{n_i}{N_i} \end{aligned}$$

where $n_i$ is the number of correctly classified utterances in the $i$-th class, $N_i$ is the total number of utterances in the $i$-th class, and $k$ is the number of emotion classes.

## Results and analysis

In this section, we evaluate the performance of our model on different datasets (IEMOCAP, EMODB, EMOVO) through model comparison experiments. Ablation studies are meticulously designed to further investigate the effectiveness of the MS-EmoBoost strategy and assess the importance of its key components. Additionally, we conduct generalization experiment to ascertain the robustness of the MS-EmoBoost strategy across diverse self-supervised models.

### Results and comparison

Table 2 provides a comprehensive comparison of various models on the IEMOCAP, EMODB and EMOVO datasets, highlighting the significant contributions of our MS-EmoBoost model in the field of SER. The table categorizes the models by year and details their performance in terms of WA and UA.

**Table 2:** Comparison with the state-of-the-art systems in terms of WA (%) and UA (%).

Year	References	Model	Dataset	WA (%)	UA (%)
2022	Yue et al.^47^	MTL$_{\text {emo+int}}$	IEMOCAP	68.29	70.82
2021	Wang et al.^48^	EF-w2v-base	IEMOCAP	70.75	-
2024	Striletchi et al.^49^	TBDM-Net::BT	IEMOCAP	70.05	71.78
2023	Ye et al.^50^	TIM-NET	IEMOCAP	71.65	72.50
2022	Zou et al.^18^	Co-attention	IEMOCAP	71.64	72.70
2024	**Ours**	**MS-EmoBoost**	IEMOCAP	**72.10**	**72.91**
2023	Mihalache et al.^51^	FCNNS	EMODB	82.9	82.6
2024	Goel et al.^52^	CAMuLeNet	EMODB	86.2	-
2024	Striletchi et al.^49^	TBDM-Net::BT	EMODB	88.23	90.01
2023	Liu et al.^53^	Cascaded Attention Network	EMODB	91.58	88.76
2023	Chauhan et al.^54^	CwGHP	EMODB	90.81	92.59
2024	**Ours**	**MS-EmoBoost**	EMODB	**92.45**	**92.62**
2023	Ma et al.^55^	emotion2vec	EMOVO	61.21	62.97
2021	Tuncer et al.^56^	TSP+INCA	EMOVO	79.08	79.08
2024	Striletchi et al.^49^	TBDM-Net::BT	EMOVO	82.12	84.20
2022	Wen et al.^57^	CPAC	EMOVO	85.40	85.40
2024	**Ours**	**MS-EmoBoost**	EMOVO	**86.88**	**87.51**

Significant values are given in bold.

Specifically, our MS-EmoBoost strategy achieved 72.10% WA and 72.91% UA on the IEMOCAP dataset, effectively addressing the shortcomings in emotional information representations within self-supervised features. It significantly surpasses methods that employ multi-task learning (MTLemo+int^47^), entirely fine-tuning (EF-w2v-base^48^), and multi-acoustic features fusion (Co-attention^18^). Moreover, MS-EmoBoost achieved outstanding results on the EMODB dataset, with 92.45% WA and 92.62% UA, as well as 86.88% WA and 87.51% UA on the EMOVO dataset. These results not only highlight its strong adaptability across different emotional speech databases but also its remarkable ability to accurately capture and classify a wide range of emotional states.

Overall, the MS-EmoBoost strategy has made substantial advancements in enhancing self-supervised speech emotion expressions, demonstrating the potential of self-supervised learning paradigms to improve the efficiency of SER systems. This establishes a promising direction for future research to explore further improvements and applications in diverse real-world scenarios.

**Figure 4:**
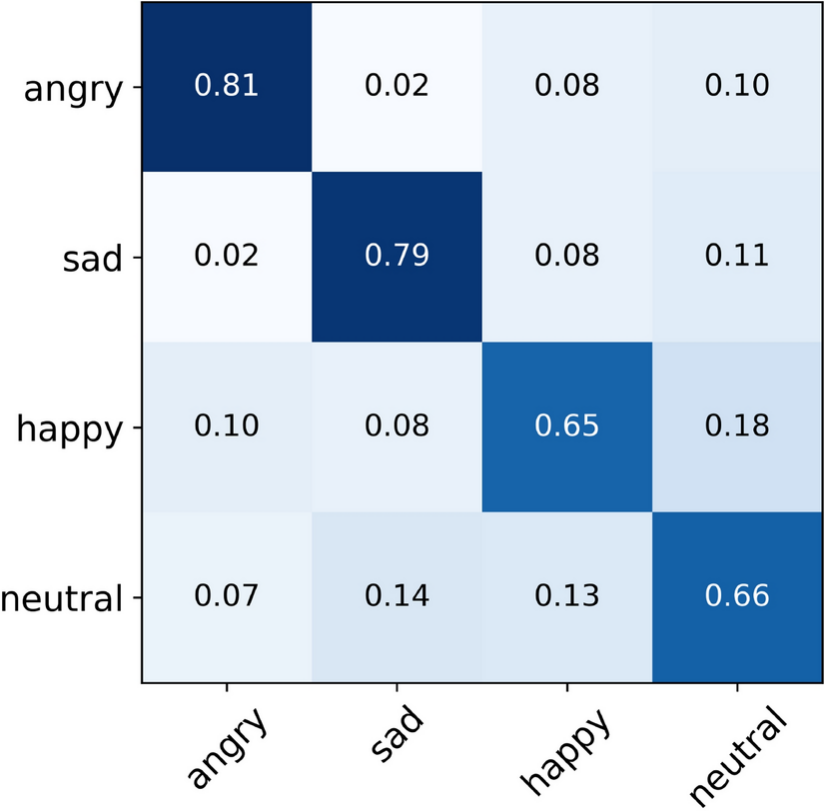
Final normalised confusion matrix of MS-EmoBoost on the IEMOCAP dataset.

The confusion matrix in Fig. 4 displays the performance of our method on the IEOMCAP dataset across four emotional categories. Observations indicate that the system generally performs well on the IEMOCAP dataset, particularly in recognizing the emotions of anger and sadness. However, it exhibits weaker performance in identifying happy and neutral emotions. A significant confusion between happy and neutral categories is noted, particularly with many happy samples being misidentified as neutral. This issue may stem from the abundant sample data for happy and neutral emotions, which could lead the model to learn non-representative features and noise during the training process.

**Figure 5:**
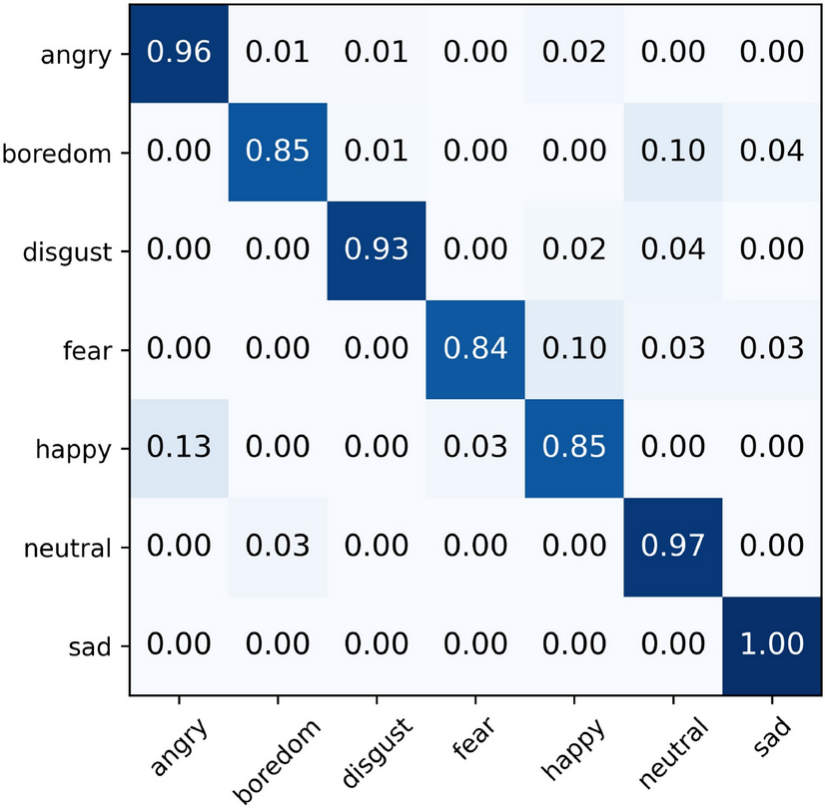
Final normalised confusion matrix of MS-EmoBoost on the EMODB dataset.

**Figure 6:**
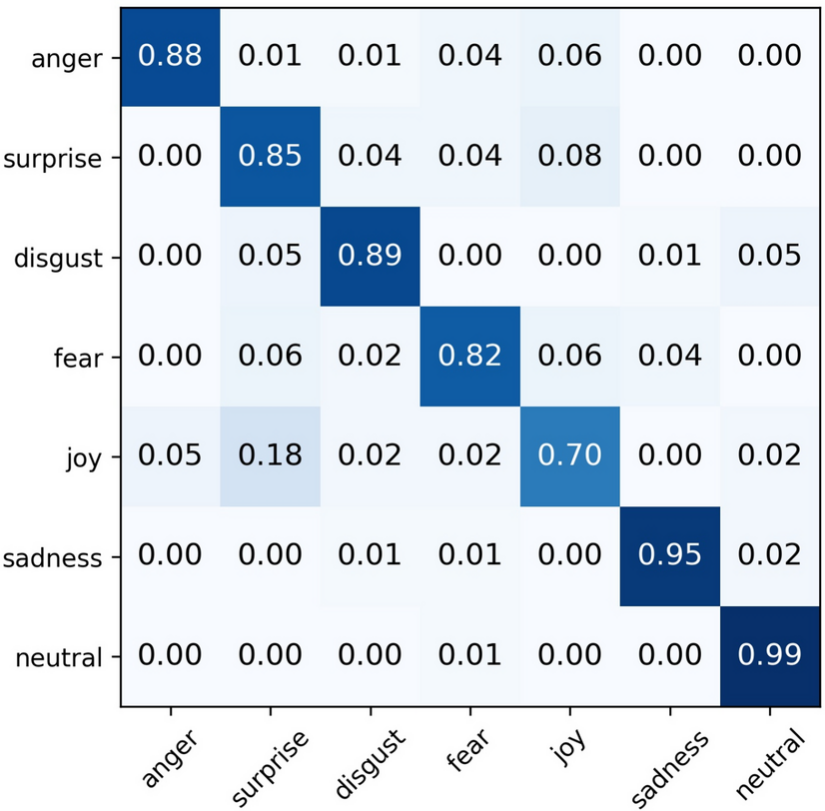
Final normalised confusion matrix of MS-EmoBoost on the EMOVO dataset.

The confusion matrix results (Figs. 5 and 6) demonstrate the emotion recognition performance of the proposed method on the two datasets. Experiments indicate that the model achieves excellent overall performance on the EMODB dataset, with nearly perfect recognition accuracy for anger, sadness, and neutral emotions. However, significant confusion is observed between happiness and anger as well as between fear and happiness on this dataset. Similarly, in the EMOVO dataset, high recognition accuracy is achieved for all emotion categories except joy, with recognition accuracy for sadness and neutral emotions exceeding 95%. Nevertheless, a notable misclassification phenomenon is observed between happiness and surprise. These misclassifications may stem from two factors: first, the existence of similarities or overlapping regions in the acoustic feature space among different emotion categories; second, the model’s failure to adequately capture discriminative features during the feature extraction process. Therefore, future work will focus on optimizing the feature extraction module of the model by introducing more refined feature representation methods to enhance its ability to distinguish between closely related emotional states.

### Ablation study

To further validate the effectiveness of this approach, we have designed an ablation study on emotional guidance information. Additionally, to verify the significance of the attention module within the MS-EmoBoost feature enhancement strategy, an ablation experiment on the attention module has also been conducted. The ablation study was conducted on the IEMOCAP dataset.

#### Emotional guidance information

Table 3 presents the results of ablation experiments on emotional guidance information. The baseline model using only wav2vec 2.0 Base without emotional guidance information exhibits relatively lower accuracy. This suggests that while wav2vec 2.0 Base possesses strong capabilities in extracting speech features, it may not sufficiently capture emotional details in SER tasks. Upon separately considering MFCC and spectrogram as guiding features for the baseline model, WA and UA showed significant improvements, each increased by at least 5 percentage points. This indicates that emotional information within self-supervised features was highlighted and complemented. Furthermore, when considering both MFCC and spectrogram in conjunction, employing the MS-EmoBoost strategy yielded optimal performance, with WA and (UA reaching 72.1% and 72.91%, respectively. Compared to the baseline model, this shows improvements of 8.07% and 7.24%, respectively.

**Table 3:** Ablation experiment results of emotional guidance information.

Model	WA (%)	UA (%)
baseline (wav2vec 2.0 Base)^18^	64.03	65.67
wav2vec 2.0 Base+MFCC	70.69	71.43
wav2vec 2.0 Base+Spectrogram	69.93	71.74
**wav2vec 2.0 Base+MFCC+Spectrogram**	**72.10**	**72.91**

Significant values are given in bold.

**Figure 7:**
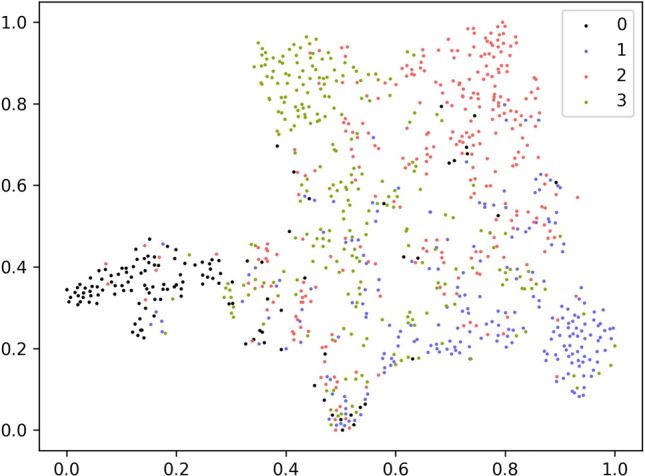
wav2vec 2.0 base.

**Figure 8:**
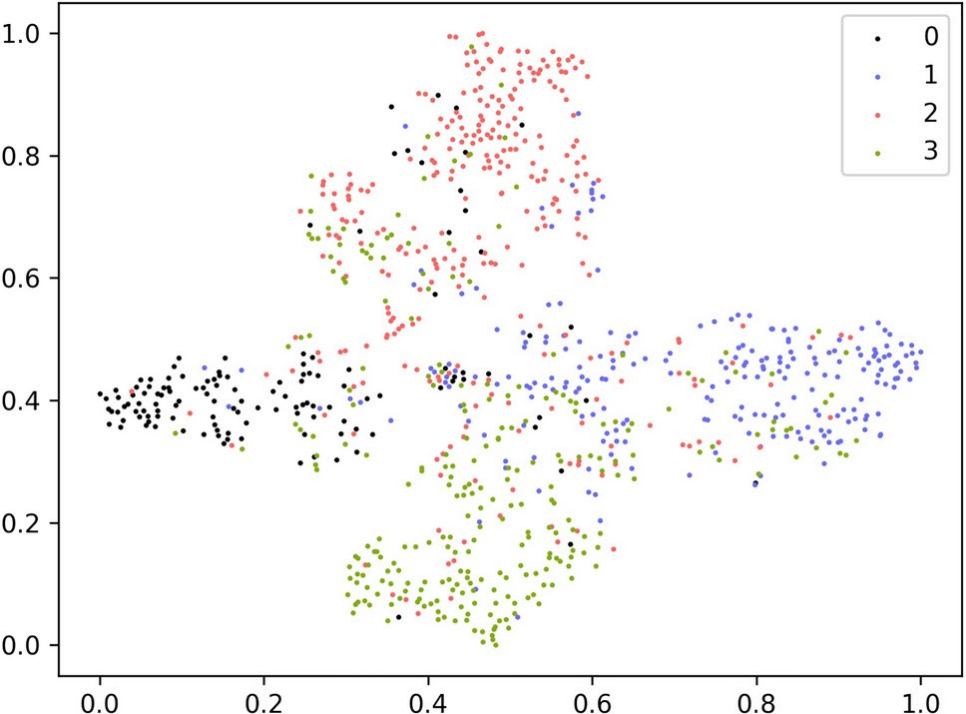
wav2vec 2.0 Base w/MS-EmoBoost.

Figures 7 and 8 display the t-SNE visualizations of wav2vec 2.0 Base features with and without MS-EmoBoost, respectively. In these figures, the numbers 0, 1, 2, and 3 correspond to the four emotions: angry, sad, happy, and neutral. By comparing the two images, it can be observed that the features enhanced with the MS-EmoBoost strategy exhibit clearer classification boundaries and stronger clustering of data points within each category. This improved clustering and distinction among categories suggest that the enhanced features have greater classification abilities, enabling better performance in the final task of emotion classification.

The results of the emotional guidance information ablation experiments indicate that utilizing MFCC or spectrogram individually for guiding self-supervised feature enhancement task significantly improves the overall performance of the model. However, combining both yields the best results. This underscores the crucial role of emotional guidance information in SER task. Our proposed MS-EmoBoost feature enhancement strategy effectively enhances the performance of the wav2vec 2.0 Base features, facilitating its capability in accomplishing SER task.

#### Attention module in MS-EmoBoost

Table 4 compares of recognition results within our proposed MS-EmoBoost module with and without an attention module. The experimental results clearly demonstrate the positive impact of incorporating the attention module into the model. Without the attention module, the model achieved a WA of 67.85% and an UA of 68.95%, in contrast to 72.10% WA and 72.91% UA with attention. This improvement underscores the role of the attention module in enabling the model to focus on more crucial emotional information. The attention module aids the model in prioritizing crucial emotional information within MFCC features and spectrograms, effectively enhancing the model’s recognition performance in SER task.

**Table 4:** Ablation experiment results of attention module in MS-EmoBoost.

Attention module	WA (%)	UA (%)
w/o attention	67.85	68.95
**w/ Attention**	**72.10**	**72.91**

Significant values are given in bold.

### Generalization experiment

To evaluate the generalizability of our proposed MS-EmoBoost self-supervised feature enhancement strategy across different self-supervised models (model size, type, and pre-training data), we conducted experiments using three distinct models: wav2vec 2.0 Large-LS-960, HuBERT Base-LS-960, and HuBERT Large-LL-60k. These models were evaluated on the IEMOCAP, EMODB and EMOVO datasets. The experimental results are shown in Table 5.

**Table 5:** Performance of MS-EmoBoost on Other Self-Supervised Models (LS-960 denotes the model pre-trained on the 960-hour English LibriSpeech dataset; LL-60k indicates the model pre-trained on the 60,000-hour English Libri-Light^58^ dataset).

Model	IEMOCAP WA (%)	IEMOCAP UA (%)	EMODB WA (%)	EMODB UA (%)	EMOVO WA (%)	EMOVO UA (%)
wav2vec2.0 Large-LS-960	67.36	68.62	84.62	83.76	78.77	79.20
HuBERT Base-LS-960	69.41	69.33	78.46	77.78	81.63	82.22
HuBERT Large-LL-60k	71.10	71.35	86.82	83.87	75.18	76.37
**wav2vec2.0 Large-LS-960 (w/ MS-EmoBoost)**	**71.80**	**73.92**	**92.68**	**93.06**	**82.47**	**83.31**
**HuBERT Base-LS-960 (w/ MS-EmoBoost)**	**72.58**	**73.13**	**93.92**	**94.29**	**85.21**	**85.88**
**HuBERT Large-LL-60k (w/ MS-EmoBoost)**	**73.38**	**73.08**	**93.10**	**92.52**	**77.38**	**78.36**

Significant values are given in bold.

The experimental results demonstrate that the MS-EmoBoost strategy effectively enhances the emotional expression capabilities of various self-supervised models, significantly improving their accuracy in the SER tasks across different datasets. Notably, the HuBERT Base-LS-960 model exhibited the most substantial improvement on the EMODB dataset, with WA and UA increasing by 15.46% and 16.51%, respectively. This substantial improvement fully validates the effectiveness of the MS-EmoBoost strategy.

In contrast, the HuBERT Large-LL-60k model exhibited relatively limited improvements across the three datasets, which can be primarily attributed to its inherent architectural complexity and extensive pre-training on large-scale data, enabling it to extract relatively comprehensive emotional features during the self-supervised learning phase and thus leaving minimal room for further optimization through MS-EmoBoost. Furthermore, the model’s emotional recognition accuracy on the EMOVO Italian dataset remained significantly lower than that of other models, both before and after enhancement, potentially due to linguistic disparities that hindered its full adaptation to the specific characteristics of the Italian language environment. On the IEMOCAP and EMODB datasets, the application of MS-EmoBoost resulted in the HuBERT Large-LL-60k model’s WA outperforming its UA, indicating that the enhanced model achieves higher classification accuracy in categories with larger sample sizes.

In summary, MS-EmoBoost not only effectively enhances the performance of various SSL models across different languages in SER tasks but also reveals the intricate relationship between model complexity, pre-training data scale, and the effects of feature enhancement. These findings provide valuable insights for further research in the field of self-supervised learning.

## Conclusion and future work

In this study, we propose MS-EmoBoost, which effectively addresses the insufficient sensitivity issue of self-supervised features in capturing emotional information. By leveraging the emotional information in MFCC and spectrogram, MS-EmoBoost enhances the emotional representation capabilities of self-supervised features. Extensive experiments conducted on various self-supervised models such as wav2vec 2.0 and HuBERT confirm the effectiveness and generalization capability of the MS-EmoBoost strategy. The results on the IEMOCAP, EMODB, and EMOVO datasets demonstrate significant improvements in WA and UA metrics across all tested models. Furthermore, ablation studies on the IEMOCAP dataset underscore the pivotal roles played by attention modules and emotional guidance information, synergistically contributing to the superior performance of our approach. In summary, the MS-EmoBoost strategy demonstrates its extensive application potential in SER tasks.

The performance of the MS-EmoBoost strategy relies on the quality of emotional guidance information contained in MFCC and spectrogram. In real-world settings, speech data often includes noise and other distortions, which can undermine the effectiveness of SSL feature enhancement, thus affecting the accuracy of emotion recognition. To address these challenges, future research will focus on several key areas: First, given the sensitivity of MFCC and spectrogram to window length, we plan to evaluate the impact of different window lengths on the emotion recognition task using multiple datasets. Secondly, we will integrate acoustic information from diverse emotional layers and explore effective methods for merging deep emotional insights. Additionally, we will investigate the feasibility and strategies of enhancing SSL features with additional modal information. Considering the high computational demands of the SSL-based emotion recognition framework, which may hinder deployment on resource-limited devices, we will also evaluate the feasibility of model compression and pruning techniques to facilitate real-time applications on edge devices.

## Data Availability

The IEMOCAP dataset is publicly available at https://sail.usc.edu/iemocap/index.html. The EMOVO dataset is publicly accessible at https://www.kaggle.com/datasets/sourabhy/emovo-italian-ser-dataset/data. Additionally, the EMODB dataset is available at http://emodb.bilderbar.info/start.html. All data generated and analyzed during this study are included in this published article.
